# Dynamic Causal Modeling for fMRI With Wilson-Cowan-Based Neuronal Equations

**DOI:** 10.3389/fnins.2020.593867

**Published:** 2020-11-27

**Authors:** Sadjad Sadeghi, Daniela Mier, Martin F. Gerchen, Stephanie N. L. Schmidt, Joachim Hass

**Affiliations:** ^1^Department of Theoretical Neuroscience, Central Institute of Mental Health, University of Heidelberg/Medical Faculty Mannheim, Mannheim, Germany; ^2^Bernstein Center for Computational Neuroscience (BCCN) Heidelberg/Mannheim, Mannheim, Germany; ^3^Department of Physics and Astronomy, University of Heidelberg, Heidelberg, Germany; ^4^Department of Clinical Psychology, Central Institute of Mental Health, University of Heidelberg/Medical Faculty Mannheim, Mannheim, Germany; ^5^Department of Psychology, University of Konstanz, Konstanz, Germany; ^6^Faculty of Applied Psychology, SRH University of Applied Sciences Heidelberg, Heidelberg, Germany

**Keywords:** dynamical causal modeling, fMRI, Bayesian model selection, Wilson-Cowan equation, effective connectivity, mirror neuron system

## Abstract

Dynamic causal modeling (DCM) is an analysis technique that has been successfully used to infer about directed connectivity between brain regions based on imaging data such as functional magnetic resonance imaging (fMRI). Most variants of DCM for fMRI rely on a simple bilinear differential equation for neural activation, making it difficult to interpret the results in terms of local neural dynamics. In this work, we introduce a modification to DCM for fMRI by replacing the bilinear equation with a non-linear Wilson-Cowan based equation and use Bayesian Model Comparison (BMC) to show that this modification improves the model evidences. Improved model evidence of the non-linear model is shown for our empirical data (imitation of facial expressions) and validated by synthetic data as well as an empirical test dataset (attention to visual motion) used in previous foundational papers. For our empirical data, we conduct the analysis for a group of 42 healthy participants who performed an imitation task, activating regions putatively containing the human mirror neuron system (MNS). In this regard, we build 540 models as one family for comparing the standard bilinear with the modified Wilson-Cowan models on the family-level. Using this modification, we can interpret the sigmoid transfer function as an averaged f-I curve of many neurons in a single region with a sigmoidal format. In this way, we can make a direct inference from the macroscopic model to detailed microscopic models. The new DCM variant shows superior model evidence on all tested data sets.

## Introduction

Since its invention, functional magnetic resonance imaging (fMRI) has been developed into a powerful and versatile measurement technique. Apart from localizing a wide range of brain functions, it can now also be used to make statistical inferences about the neural network underlying these functions. This kind of inference has been made possible by sophisticated analysis techniques such as dynamic causal modeling (DCM). DCM is a well-established method to investigate the causal structure (effective connectivity) of a system of brain regions. It uses a Bayesian framework to deduce hidden neuronal states from time series of observed data measured by fMRI or other neuroimaging tools such as electroencephalography (EEG) or magnetoencephalography (MEG). DCM provides posterior estimates of intrinsic synaptic coupling strengths among neuronal populations, the inputs that modulate those couplings, and extrinsic inputs driving the neuronal states (Friston et al., 2003; David et al., 2006).

The interpretability of DCM is limited by the expressiveness or complexity of the underlying neural model. This complexity is constrained by the nature of the data at hand. As we will see below, the best generalizing models have the most significant model evidence. Log model evidence is accuracy minus complexity. This means that there is an optimal model complexity for any given kind of data. In what follows, we ask whether typical fMRI data could support more expressive or complex models that incorporate sigmoid activation functions, which are characteristic of neuronal dynamics. The most current versions of DCM for fMRI rely on a relatively simple, completely linear model of neuronal activity, which is justified as a Taylor expansion of more complex dynamics near a fixed point (Friston et al., 2003, 2014; Marreiros et al., 2008a; Stephan et al., 2008; Daunizeau et al., 2009). This is mainly due to the low temporal resolution of the fMRI data, making it necessary to estimate parameters from a very limited number of data points and restricts the number of parameters that can reasonably be inferred (David et al., 2008). DCM has also been used in EEG and MEG with considerably more complex neuronal state equations than in standard bilinear DCM for fMRI (David et al., 2006; Kiebel et al., 2008; Moran et al., 2013), as the finer time resolution allows to constrain a wider range of neuronal processes at different time scales. Very recently, more complex models have also been applied to fMRI data, including simulated superficial and deep pyramidal cells, spiny-stellate excitatory and inhibitory interneurons, all contributing to the ongoing dynamics (Friston et al., 2019; Jafarian et al., 2020; Wei et al., 2020) (for a more detailed comparison of the existing DCM variants, see section “Comparison to other DCM extensions” in the Discussion).

While the increased complexity of such extended DCMs opens the possibility to make more detailed inference about the networks underlying brain functions, it also makes those models harder to fit the data, as increasing the number of fitted parameters increases both computational cost and the risk of obtaining suboptimal fits. Furthermore, it has been shown in other contexts that complex models with a large number of parameters can be seriously underconstrained, i.e., several qualitatively different sets of parameters fit the data equally well, making it hard to interpret the results (Marder and Prinz, 2002). Thus, fitting of complex models to data with limited resolution can result in solutions that produce good fits, but unphysiological parameter regimes. Even worse, fitting may result in physiologically plausible solutions, which point toward neural mechanisms that are nevertheless entirely different from those being used by the brain.

We propose a solution to the dilemma between detailed inference and underconstrained modeling using a DCM, which is relatively simple but involves a more realistic, non-linear neuron model. More precisely, Wilson-Cowan-type equations (Wilson and Cowan, 1972), which describe the evolution of excitatory and inhibitory activity in a population of neurons, are implemented instead of standard bilinear equations for both single and two-state DCM. In this way, the parameters obtained by DCM can be directly interpreted physiologically (see “Materials and Methods” section). In the future, these DCM results can be used to constrain a spiking network model (Hass et al., 2016) to derive predictions about physiological details that cannot be obtained from non-invasive recordings.

We test the new non-linear modification of the DCM framework based on the Wilson-Cowan model (W-C DCM) on three different data sets and show its superiority in model evidences compared with the standard bilinear model. First, we use an established data set that has been widely used as a test case for DCM (Christian Büchel and Friston, 1997). This section compares W-C DCM with the bilinear DCM for the two best models achieved from previous studies (Penny et al., 2004a; Ashburner et al., 2014). Second, we investigate the dynamics of the human mirror neuron system (MNS) using our own novel data set. Here, W-C DCM is shown to unravel connections which are overlooked by bilinear DCM. Finally, we generate synthetic data with different signal-to-noise ratios (SNRs) based on the novel data to investigate how W-C DCM performs when the ground truth is known. We show that W-C DCM provides explanations with more significant evidence compared to bilinear DCM for low SNRs, which are typical for fMRI data.

## Materials and Methods

### Wilson-Cowan Equations for DCM

In this section, we briefly review single-state Dynamic Causal Modeling for fMRI data (Friston et al., 2003) as well as the Wilson-Cowan model (Wilson and Cowan, 1973, 1972) before introducing the modifications of the neuronal state equation.

DCM describes a system characterized by *m* inputs and *l* outputs with one output in each brain region. The experimental manipulations are modeled as changes in the inputs. In each of these regions, the output is measured, which corresponds to the observed BOLD signal. Normally these time series are considered as average or typical values of given brain regions. Each region is described by five state variables, four of which correspond to the hemodynamic model, i.e., the dilatation of the blood vessels, the normalized blood flow, the normalized venous volume, and the deoxyhemoglobin content of the blood (Friston et al., 2000; Stephan et al., 2007b). These variables are independent of the state of other brain regions. The fifth state variable is the neuronal or synaptic activity in each brain region, modulated by the neuronal states in other regions.

The effective connectivity of the regions is described at the neuronal level. This neuronal activity is modeled by a multivariate differential equation that has a bilinear form in the original format (Friston et al., 2003) to describe the dynamics:

(1) $${\text{ ˙z}}_{t}=\left(A+\sum_{j=1}^{m}u_{j}\,B^{j}\right)\,z_{t}+\mathrm{Cu}$$

*z*_*t*_ denotes the time derivative of neuronal activity (*z*) and *u*_*j*_ is the j-th of m inputs at time t. Matrix A, also called the connectivity matrix, describes the interconnections between the brain regions and the influence that a neural system exerts on another. The matrices *B*^*j*^ describe the change in connectivity through the j-th modulatory input *u*_*j*_. Finally, the matrix C embodies the direct influences of the external inputs *u* on the neuronal activity. This equation can be achieved from a Taylor expansion of any non-linear function, *F*(*z*,*u*,θ), around the system’s resting state (*z* = 0,*u* = 0). Such a non-linear function can be thought to describe both the synaptic transmission between regions and the neural computations within each region. Thus, when estimating the connectivity matrices, θ = {*A*,*B*^*j*^,*C*}, the estimated numbers also reflect neural computations and synaptic transmission together. Thus, one goal of the presented framework based on the Wilson-Cowan model is to disentangle local computation (using a non-linear transmission function S) and transmission between regions (using the linear connectivity matrices θ = {*A*,*B*^*j*^,*C*}, which thus are to be interpreted differently compared to the bilinear model).

The Wilson-Cowan model describes the evolution of firing rates of a large population of densely coupled neurons. Assuming both excitatory (E) and inhibitory neurons (I) in this population to be homogeneous, their firing rates *R*_*E*_(*t*) and *R*_*I*_(*t*) are governed by two differential equations:

(2) $$\begin{array}{c} \tau_{E}\,{\dot{R}}_{E}\,\left(t\right)=-R_{E}\,\left(t\right)+S_{E}\,\left(x_{E}\right)\\ \tau_{I}\,{\dot{R}}_{I}\,\left(t\right)=-R_{I}\,\left(t\right)+S_{I}\,\left(x_{I}\right)\\ S_{E}\,\left(x_{E}\right)=\frac{1}{1+\exp\,\left(-\alpha_{E}*\left(x_{E}-\theta_{E}\right)\right)}\\ S_{I}\,\left(x_{I}\right)=\frac{1}{1+\exp\,\left(-\alpha_{I}*\left(x_{I}-\theta_{I}\right)\right)}\\ x_{E}=w_{\mathrm{EE}}*R_{E}\,\left(t\right)-w_{\mathrm{EI}}*R_{I}\,\left(t\right)+I_{E}\\ x_{I}=w_{\mathrm{IE}}*R_{E}\,\left(t\right)-w_{\mathrm{II}}*R_{I}\,\left(t\right)+I_{I} \end{array}$$

**FIGURE 1:**
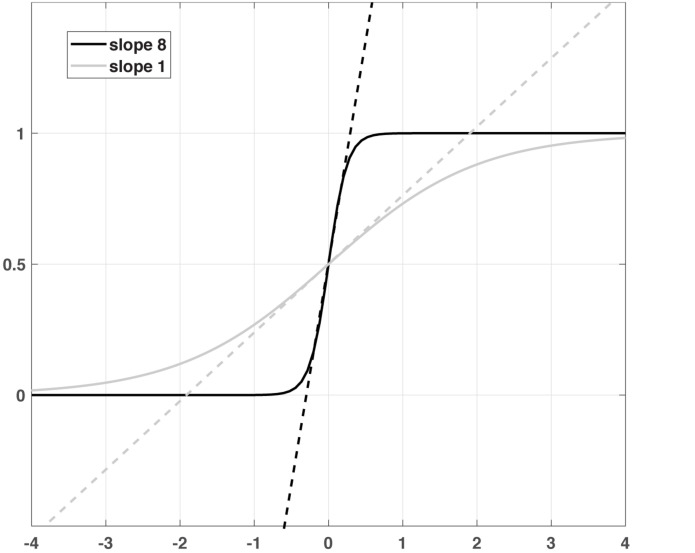
A schematic illustration of two sigmoid functions with different slopes and the corresponding linear functions.

τ_*E*_ and τ_*I*_ are the membrane time constants of the two subpopulations, and *S*(*x*) denotes the sigmoidal non-linearity as an activation or transfer function with slope α and threshold θ, which are also specific for E and I. *w*_*XY*_ is the synaptic weight of the connection from subpopulation X to Y and *I*_*X*_ represents the external input to each subpopulation, where X and Y can be E or I. The first differential equation describes an exponential relaxation of the firing rate *R*_*E*_(*t*) with time constant τ_*E*_ to its steady-state value *S*_*E*_(*x*_*E*_), which is determined by a weighted sum *x*_*E*_ of both firing rates *R*_*E*_(*t*) and *R*_*I*_(*t*) as well as the external input *I*_*E*_, filtered by the sigmoid non-linearity *S*_*E*_. The same is true for *R*_*I*_(*t*) with its respective variables and parameters. The weights in the sum can be directly interpreted as synaptic efficiencies between the subpopulations, while the sigmoid mimics the non-linear input-output relations of the neurons in the subpopulation (Figure 1). For large values α, this relation is very steep, so *S* is zero for inputs *x* below the threshold θ and one for input above. The relation becomes more gradual for lower slopes, but still saturates into zero and one for very low and very high inputs, respectively.

Wilson and Cowan used phase-plane analysis to show that the system described by Eq. 2 allows for a variety of dynamic phenomena that are relevant to the function of the brain (Wilson and Cowan, 1972), including multiple stable fixed points (a simple mechanism e.g., for working memory) and oscillations.

We propose to replace the standard bilinear equation (equation 1) with a Wilson-Cowan-type equation:

(3) $$\begin{array}{c} {\text{ ˙z}}_{t}=-z_{t}+S\,\left(x\right)\\ S\,\left(x\right)=\frac{1}{1+\exp\,\left(-\alpha *x\right)}-\frac{1}{2}\\ x=\left(A+\sum_{j=1}^{m}u_{j}\,B^{j}\right)\,z_{t}+\mathrm{Cu} \end{array}$$

In this way, the different components of the model relate to the underlying biological elements of the brain: The matrices A, B, and C are the synaptic weights (*w*) in Eq. 2 (parameters merge excitatory and inhibitory synaptic weights), and the sigmoid non-linearity directly relates to the f-I curve of single neurons (Hass et al., 2016). This contrasts with the bilinear model, where the matrices intermingle synaptic weights and Taylor approximations of the non-linearities. Note that this implementation of the sigmoid function allows for negative firing rates to ensure the neuronal system has a stable fixed point when all states are equal to zero and changes in state variable can be interpreted as deviations from the fixed point (cf. David et al., 2006). As a negative firing rate is not physiologically plausible, the model can be interpreted to capture activity relative to a baseline resting-state rather than total activity.

In this form, we replace separate excitatory and inhibitory variables with a single neuronal variable that can be positive or negative. However, it is possible to consider separate excitatory and inhibitory variables explicitly, using an extension of the two-state DCM.

### Two-State DCM With Wilson-Cowan Equations

We also incorporated the Wilson-Cowan model (Eq. 2) into two-state DCM (Marreiros et al., 2008a) to compare its application between single- and two-state models and use it in the future to constrain local spiking neural networks in a more detailed way. In this version of DCM, each region consists of excitatory and inhibitory subpopulations, and in this way, it is biologically more plausible and less constrained than the single state model. This form of the DCM is more similar to the original Wilson-Cowan model (equation 2, Wilson and Cowan, 1972, 1973). The standard bilinear equations of a two-state model are:

(4) $$\begin{array}{c} {\dot{x}}_{E}=w_{\mathrm{EE}}\,x_{E}-w_{\mathrm{SE}}\,x_{E}-w_{\mathrm{IE}}\,x_{I}+\mathrm{Cu}\\ {\dot{x}}_{I}=w_{\mathrm{EI}}\,x_{E}-w_{\mathrm{SI}}\,x_{I} \end{array}$$

Similar to the W-C model, the *x*_*E*_ and *x*_*I*_ summarize the dynamics of the excitatory and inhibitory neurons. w_EE_ is the extrinsic connection between excitatory neurons of different regions (The connections between the two regions are provided only by the excitatory neurons), and w_IE_ and w_EI_ are the within region (intrinsic) connections from excitatory (E) to inhibitory (I) populations and vice versa. Finally, w_SI_ and w_SE_ represent the intrinsic inhibitory self-connection on I, and excitatory self-connection on E, respectively. Due to the difference between intrinsic and extrinsic connections, x_E_ has two different meanings in Eq. 4: In the term including w_EE_, x_E_ represents extrinsic input from different brain regions, while in all other terms, intrinsic input from the same region is meant. Furthermore, in this formulation, the between regions connections (*w*_*EE*_) and intrinsic inhibitory to excitatory connections (w_IE_) are split up into a direct and modulatory part, analogous to the A and B matrix components for single-state DCM.

For excitatory and inhibitory subpopulations, we modified these equations as below:

(5) $$\begin{array}{c} {\dot{z}}_{E}=-z_{E}+S_{E}\,\left(x_{E}\right)\\ {\dot{z}}_{I}=-z_{I}+S_{I}\,\left(x_{I}\right)\\ S_{E}\,\left(x_{E}\right)=\frac{1}{1+\exp\,\left(-\alpha_{E}*x_{E}\right)}-\frac{1}{2}\\ S_{I}\,\left(x_{I}\right)=\frac{1}{1+\exp\left(-\alpha_{I}*x_{I}\right)}-\frac{1}{2}\\ x_{E}=w_{\mathrm{EE}}\,z_{E}+w_{\mathrm{SE}}\,z_{E}-w_{\mathrm{IE}}\,z_{I}+\mathrm{Cu}\\ x_{I}=w_{\mathrm{EI}}\,z_{E}-w_{\mathrm{SI}}\,z_{I} \end{array}$$

Where α_*E*_ and α_*I*_ are the slope of sigmoid function in the excitatory and inhibitory subpopulations. In the original model in SPM (Marreiros et al., 2008a), only w_EE_ and w_IE_ are estimated, but here we estimate all the parameters as well as the sigmoidal slopes for excitatory and inhibitory neurons.

### Bayesian Model Selection

We use Bayesian Model Selection (BMS) for comparing the Wilson-Cowan-based equations with bilinear DCMs. Bayesian Model Selection is widely used for finding the model that fits the data best among several alternatives. Model evidence (the probability of obtaining observed data given the model) is widely used in this approach, using the free-energy criterion. This criterion is composed of two components: the accuracy term (log-likelihood of data), which computes the data fit, and the complexity term, which depends on the number of parameters and also the deviation of posterior densities from their prior. Two models *m*_*1*_ and *m*_*2*_ are compared using the Bayes Factor (BF_12_), which is the ratio of model evidence of two models reported on a log scale. Its value equals the difference between the free energy of the models (|*F*_1_−*F*_2_|). By convention, if the value of the log-*BF*_*12*_ is about three or more, it indicates strong evidence in favor of model 1 over 2 (Kass and Raftery, 1995; Raftery, 1995).

There are two different approaches at the group level for model inference: Fixed Effects (FFX) and Random Effects (RFX) analysis. In the FFX, Group Bayes Factors (GBF) (Stephan et al., 2007a) are widely used for model selection when a common model is being assumed for each subject, i.e., the most likely model structure is the same across subjects (Stephan et al., 2009; Penny et al., 2010). This method is sensitive to outliers and blind concerning group heterogeneity. Hierarchical Random Effects analysis (RFX), on the other hand, models inference on the level of group analysis that allows each subject to have a different best model and computes the probability of all subjects’ data given each model. In contrast to FFX, outliers have minimal effect on RFX results, which accounts for group heterogeneity. The results of RFX group analyses are reported in terms of expected, exceedance, and protected exceedance probabilities. The expected probability is the expected posterior probability of obtaining the n-th model for any randomly selected subject, and the exceedance probability is the probability that one model is more likely than any other model between all models tested. As the exceedance probability does not consider the null hypothesis that all model frequencies are due to chance, the protected exceedance probability is also utilized here (Rigoux et al., 2014), which considers this null hypothesis. Each of these measures can be used for finding the best model, and higher expected, exceedance, or protected exceedance probability independently means that a model is more probable. However, fixed effects BMS is also used in this study to show that the modified version of DCM has a better result in both Random and Fixed effects analysis. Furthermore, for the established data set (Büchel and Friston, 1997), as it is only for one subject, FFX BMS usage for testing our modification is mandatory.

In this study, we performed the BMS on the family-level (Penny et al., 2010) and grouped all the possible models in one family to compare the Bilinear and W-C DCMs. The implementation was originally developed based on DCM10 (r6313) provided with SPM8 (Statistical Parametric Mapping 8)^1^, which was the most recent version when this work was begun. However, we repeated several analyses with DCM12 (r7487) in SPM12 and did not observe any qualitative differences. Furthermore, we computed the protected exceedance probabilities with the VBA toolbox as it is not implemented in the SPM software at the family level (Daunizeau et al., 2014; Rigoux et al., 2014).

Moreover, to characterize the two models at the microcircuitry level, we perform Bayesian Model Averaging (BMA). This method averages each connectivity parameter over all models within the family or whole model space, weighted by each models’ posterior probabilities. Thus, the most probable models will contribute the most to the model averaging (Penny et al., 2010).

In order to have a fair comparison between the Bilinear and W-C DCMs, we used the original format of shrinkage priors and the identical hyperpriors as the classical DCM (Friston et al., 2003; Marreiros et al., 2008a) for both single and two-state W-C DCMs. Furthermore, we also used the same inference Variational Bayes under the Laplace assumption (VBL) scheme as the original DCM (Friston et al., 2007).

### Data Sets

#### Established Data Set

We used well-studied data from an experiment on visual attention-modulated connectivity during visual motion processing (available from the SPM website: http://www.fil.ion. ucl.ac.uk/spm, the full description of the experimental paradigm can be found in Büchel and Friston, 1997). In brief, the experimental variables were three exogenous inputs: A “photic stimulation” variable indicated when dots were shown on a screen, a “motion” variable indicated that the dots were moving, and the “attention” variable indicated that the subject should attend to possible velocity changes. These are also the three input variables that we used in the DCM analyses shown in Figure 3A. This data set for a single subject has been used several times to validate DCM for fMRI (Harrison et al., 2003; Penny et al., 2004a, b; Marreiros et al., 2008a; Stephan et al., 2008; Friston et al., 2019).

#### Novel Empirical Data on Imitation

Empirical data were acquired within the framework of a larger project on the human MNS. Participants underwent a simultaneous EEG-fMRI measurement. Here data of the fMRI measurement is presented. The reported analyses are conducted on a subset of 42 healthy participants out of the total final sample of 75 participants that were available by the time the analyses were conducted. The study was approved by the local ethics board at the Medical Faculty Mannheim, University of Heidelberg (2015-501N-MA), and participants signed written informed consent before participating in the study.

**FIGURE 2:**
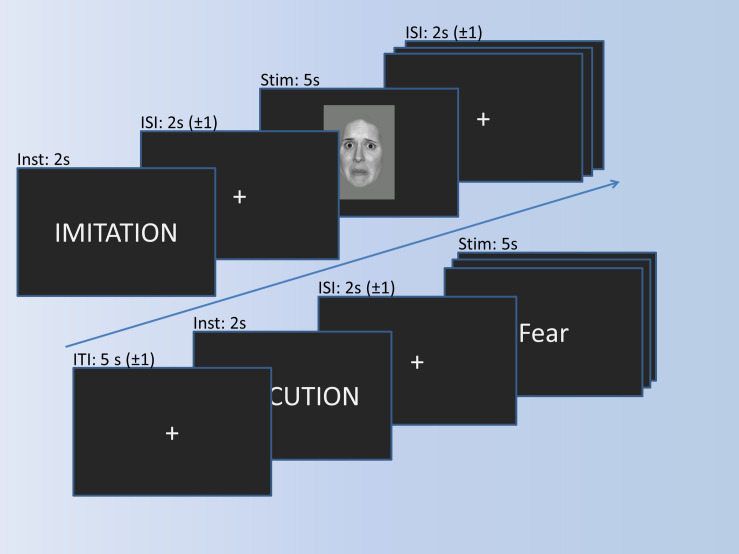
Imitation paradigm with the timing of trials. A trial for imitation and a trial for execution is shown exemplarily.

The imitation paradigm in Figure 2 consists of three conditions (Observation, Imitation, Execution) and a motor control condition (Control). During the observation, participants simply look at emotional faces, expressing anger or fear. During imitation, participants additionally imitate the facial expression displayed in the pictures. The words “anger” or “fear” are presented in the execution condition, and participants have to mimic the corresponding facial expression. The control condition requires participants to say out loud the German letters “A” (pronounced similar to “a” in “car”) or “Ä” (pronounced similar to “a” in “anger”), which should resemble the facial expressions of fear and anger, respectively. The experimental trials are presented in blocks of four pictures. Experimental blocks are alternated with control blocks, consisting of two control stimuli. Stimuli within the blocks are presented in pseudo-randomized order and separated by a jittered inter-stimulus-interval of 1–3 s. Experimental stimuli are shown for 5 s, and the control stimuli for 3 s. Before each block, an instruction cue is presented for 2 s, preceded by a jittered inter-block-interval of 4–6 s. In total, each experimental block is presented 5 times, the control block 15 times, resulting in a total of 20 trials in each experimental condition, and 30 in the control condition.

fMRI data were acquired with a 3T Siemens Magnetom Trio Tim with a 12-channel head coil at the Central Institute of Mental Health in Mannheim, Germany. Echo-planar imaging was conducted with 32 descending 3 × 3 × 3 mm slices with 1 mm gap, TR = 2,000 ms, TE = 30 ms; flip angle = 80°, field of view = 192 mm; matrix = 64 × 64. Prior to the experiments, an anatomical sequence was recorded (TR = 1570 ms, TE = 2.75 ms; flip angle = 15°, field of view = 256 mm; matrix = 256 × 256; voxel size 1 × 1 × 1 mm).

Preprocessing consisted of slice time correction, realignment to the mean image, normalization, and resampling with 3 × 3 × 3 mm voxel size, as well as smoothing with 8 mm Gaussian kernel. First-level-analyses were achieved by general linear models with the onsets of the conditions (Imitation, Observation, Execution, and Control) and the six movement parameters from the realignment procedure as covariates. First eigenvariates of the time series of imitation >control were extracted with *p* < 0.5 without a cluster size threshold while adjusting for the activation during imitation from the regions of interest (ROI’s). The ROI’s were the main regions associated with the human MNS: BA 44, IPL, and STS. The masks for BA 44 (Brodmann atlas) and IPL (AAL atlas) were taken from the WFU_pickatlas. The BA44 mask was smoothed with a dilation factor of 1, to allow a continuous mask. The STS mask was based on activation in a study on social cognition and has been used as the region of interest in earlier publications (Mier et al., 2010a, b).

#### Synthetic Data

To validate the results from the empirical dataset (comprising 42 participants), we generated a synthetic dataset for which the network architecture and parameter values were known. We generated the synthetic fMRI data using the standard bilinear equation (Eq. 1) and the usual hemodynamic equations (Stephan et al., 2007b). Here we use a typical connectivity model from a three-area model (Figure 8B). Its network structure consists of one driving input into the first region and feedforward connections from the first region to the second and third regions, as well as a forward connection from the third region to the second region. There is also a contextual input on the forward connections from region 1 to region 3 and from region 3 to region 2. The generating parameters were also sampled from the estimated posterior values (the mean of expected values) of the previous section’s empirical data to ensure the synthetic data is realistic (reported in Figure 8B). We simulated the BOLD signal from this model and then generated the synthetic data by adding 10 realizations of normally distributed random noise for each SNR. In this way, we have simulated 10 artificial time series for each region with different signal-to-noise ratios. Then we did the parameter estimation for both Bilinear and W-C DCMs for these synthetic data with different SNRs. In this way, we could test the robustness of the analysis for varying levels of noise. We compare the two estimates using the percentage of the observed time series variance explained by the time series predicted by DCM (Zeidman et al., 2019). SNR values range between zero and 0.5, and the repetition time (TR) equals 2 s.

Usually, one uses synthetic data to establish the face validity of DCM in terms of Bayesian model comparison. In other words, one would generate data under a variety of models and then assess the evidence for the different datasets under the models used to generate the data. This creates a confusion matrix of model evidences that can be used to establish that the model generating data was recovered via Bayesian model selection. In one sense, we have already established face validity at the level of model comparison (see above).

The use of synthetic data in this section differs slightly and speaks to the robustness of model inversion instead of validity. In some circumstances—due to the non-linearity of DCM’s—there may be a failure of convergence to the global minimum of free energy. In other words, the scheme gets trapped in local minima; usually, that random fluctuations can explain all the data. This means that the predicted data responses “flat-line.” Therefore, we assessed the ability of the W-C DCM to elude local minima by showing that inversion under different levels of noise reduces the instances of “flat-lining”—as scored with the accuracy or variance explained.

## Results

In the following sections, we will test whether the Wilson-Cowan-based equations can improve the predictions of fMRI data using both empirical and synthetic data.

### Validation of Established Data Set

**FIGURE 3:**
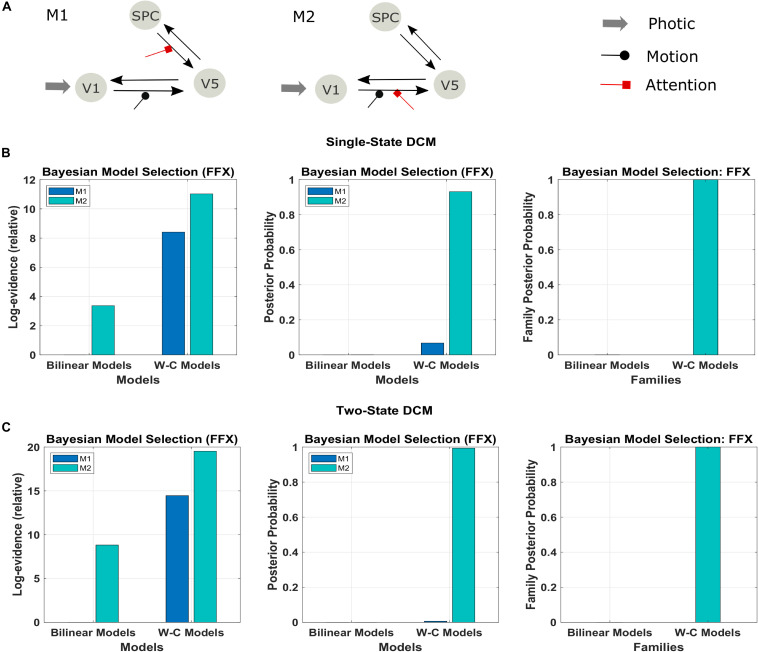
Results of the FFX Bayesian model comparison of two models of the forward and backward attention modulation (M1 and M2) for the bilinear models, and the Wilson-Cowan (W-C) models. **(A)** Illustration of the two best models (see the text). Comparison of the two models in row A for **(B)** single-state and **(C)** two-state DCM with W-C and bilinear neuronal equations (left and middle panel) and comparison of the two equation types with the two models combined in one family (right panel). The results show strong evidence (both single- and two-state) for the W-C models in all cases (family posterior probability one for W-C models and zero for standard bilinear).

In a first step, we investigate the validity of the Wilson-Cowan-based DCM framework (W-C DCM) using a well-studied data set on visual attention (Büchel and Friston, 1997). As shown in Figure 3A, activity is modeled in three regions, V1, V5, and superior parietal cortex (SPC), with sensory input to V1 and motion and attention as modulatory inputs on connections (Büchel and Friston, 1997). Previous DCM researches have established a connection scheme between these regions (Penny et al., 2004a; Marreiros et al., 2008a), so we can use this data set as a test case of our extended method.

We used the two models that had the most substantial evidence according to previous research using the Bayesian model comparison (Figure 3A; Penny et al., 2004a; Ashburner et al., 2014). The results of this comparison are represented in Figures 3B,C in terms of the relative log-evidence and posterior probability for both single- and two-state DCM. As can be seen, model 2, in which the attention input modulates the forward connection from V1 to V5, has more robust evidence in bilinear and non-linear DCM, consistent with previous findings (Marreiros et al., 2008a; Penny et al., 2004a). Furthermore, there is much stronger evidence (posterior probability) for both models in favor of Wilson-Cowan-based DCM. Thus, the modified DCM framework provides a better explanation for the data while preserving the original distinction between the two connection schemes. Please note that the dataset contains only one subject, so we performed FFX BMS, as RFX analysis can only be performed in a group analysis.

### Validation of Novel Empirical Data

Next, we apply Wilson-Cowan-based DCM to a novel data set using an imitation task to probe the human mirror neuron system (MNS), including the three regions BA44, IPL, and STS (see section “Materials and Methods” for details). For this task, it is known that the visual input goes to the STS region (Iacoboni et al., 2001; Kilner et al., 2007), and we use this hypothesis to build the model space, including 540 different models to test all the possible combinations of the forward and backward connections with their modulatory elements. We constructed the model space accordingly: From STS, the input would propagate to the IPL and BA44. The effective connectivity between the two regions can be both feed-forward or reciprocal. So in our case, we have three nodes, and these nodes can maximally have six connections in case of mutual connectivity. Furthermore, we have considered all possible modulatory inputs on the connections. In this way, each combination of the intrinsic connection between different regions can have 2^*n*^ modulatory inputs, n (in our case, n can be 2, 3, 4, 5, and 6) being the number of endogenous connections between the regions of interest. In total, for a network of three nodes and one experimental condition, one can build 5,832 (all possible models) models (Lohmann et al., 2012) (to get this number, we used the second equation in the section “Combinatorial Explosion” in the paper, *n* = 3, *m* = 1). In this way, the experimental condition (imitation in our case) can integrate into each of three regions (one region, two regions, or all three regions simultaneously; 3! = 6 different variants) and modulate the connections. However, with our hypothesis, we restricted the external input only into the STS region and could build 540 models.

**FIGURE 4:**
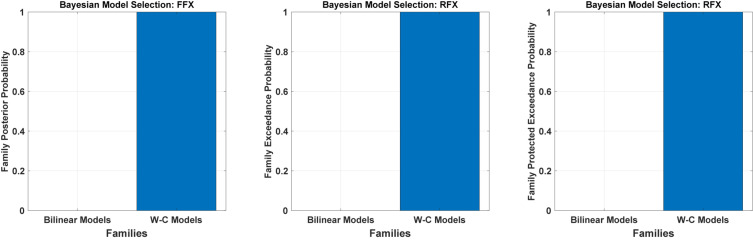
Results of Bayesian model comparisons for all possible models in the Family-level with standard bilinear equations and Wilson-Cowan (W-C) equations. This comparison is done with both FFX and RFX BMS.

We have tested all the 540 models in the family-level to compare the modified DCM to the standard bilinear DCM. As shown in Figure 4 and Table 1, the result of the Bayesian model selection for both fixed effect and random effects shows that the modified version of DCM has a probability of one and zero for the Bilinear models. For RFX BMS, the results are presented with expected, exceedance, and protected exceedance probability in Table 1. We illustrate the exceedance and protected exceedance probability in Figure 4, together with the posterior probability for FFX BMX. As can be seen, W-C models have a strong probability of one in all cases.

**TABLE 1:** RFX BMS results of the single-state and two-state models for both bilinear and Wilson-Cowan models and also the comparison between the single-state and the two-state Wilson-Cowan model (Figure 5B).

RFX BMS	Single-state model		Two-state model		Wilson-Cowan model	
	Bilinear model	Wilson-Cowan model	Bilinear model	Wilson-Cowan model	Single-state model	Two-state model
Expected probability	0.06	0.94	0.15	0.85	0.57	0.43
Exceedance probability	0	1	0	1	0.80	0.20
Protected exceedance	0	1	0	1	0.82	0.18
probability						

**FIGURE 5:**
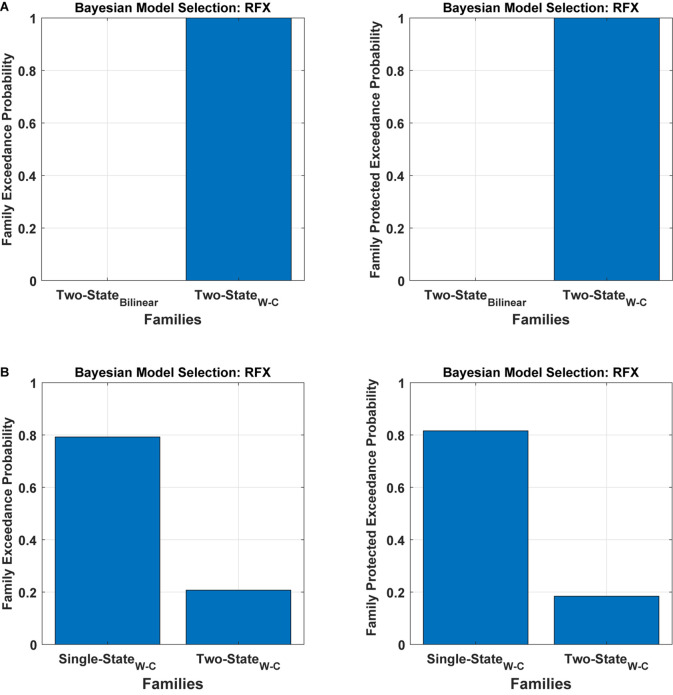
Bayesian model comparison (RFX) for the two-state model at the family-level. **(A)** comparison between two-state bilinear models and two-state Wilson-Cowan models, **(B)** comparison between single and two-state Wilson-Cowan models.

To assess the flexibility of the modification introduced above, we also applied it to two-state DCM. Figure 5 (Table 1) shows the Bayesian model comparison for two-state DCM with bilinear and Wilson-Cowan equations. In Figure 5A, by using random effects BMS, we have compared all the models on the family-level as before for the two-state DCM. As can be seen, it gives a probability of one for the modified version. In Figure 5B, we also compare the modified single- and two-state DCM with each other. In contrast to the original paper (Marreiros et al., 2008a), the single state has a higher probability with our data and thus provides a better fit to data than the two-state model. This is an important result because it shows that the best model is not necessarily the most complex model. In other words, the simpler one-state model had more evidence than the more complex two-state model (that can fit the data more accurately). We will return to this issue in the discussion.

**FIGURE 6:**
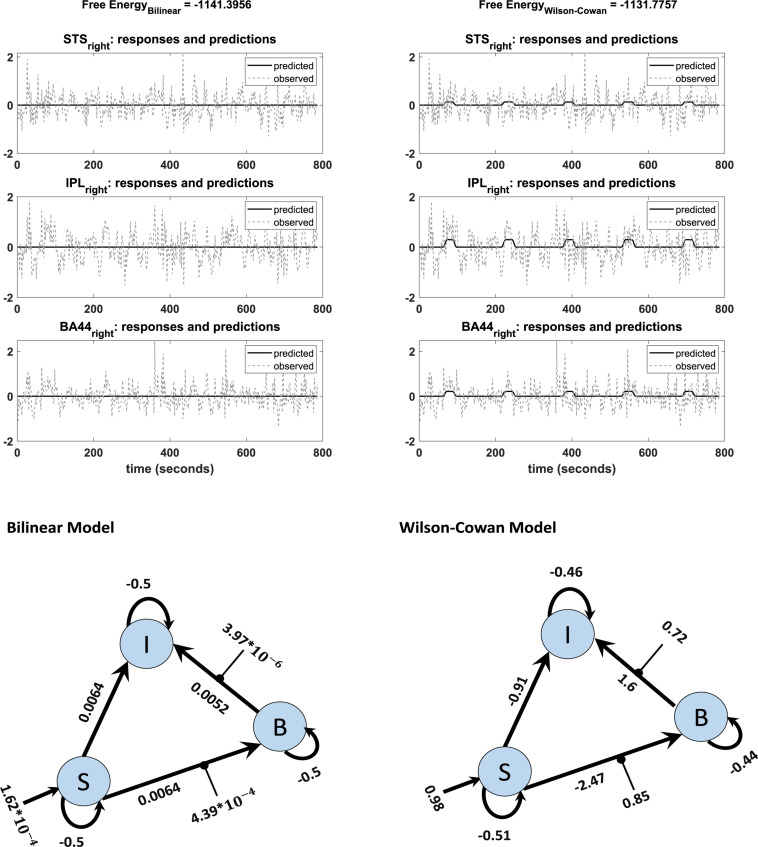
Observed and predicted time-series in DCM analysis of bilinear (left) and W-C (right) models for one subject. The left graph shows a flat time series, while in the right graph, predicted activity reacts to the inputs. The network structures with estimated parameters (the mean of expected values) of each model are illustrated below of each graph.

An additional benefit of our modification is that the Wilson-Cowan-based model produces meaningful results for participants with weak activation (e. g. showing activation for the more lenient threshold *p* = 0.5, but no activation for the stricter, standard threshold *p* = 0.05). We observed convergence to a local minimum at low activation (i.e., flat-lining) in 18 subjects out of 42 with the standard DCM, while the Wilson-Cowan-based model produced non-flat time series for almost all of these subjects (17 subjects out of 18). Figure 6 shows the differences between the outputs of DCM analysis for the two kinds of models of a typical single subject in addition to the connectivity model used to plot these graphs. Next to each connection, one can find the estimated parameters (the mean of expected values) from the Bilinear and W-C DCM. As can be seen, the W-C model’s estimated connections are much stronger than the standard bilinear model.

**FIGURE 7:**
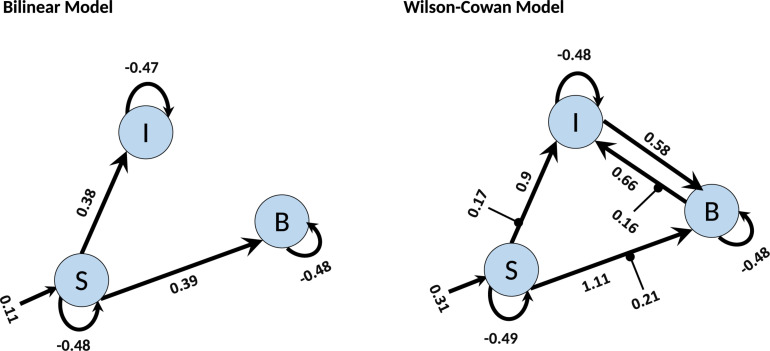
BMA results for Bilinear and W-C models. Here we illustrate only the parameters which are significantly different from zero. The values next to each connection are the expected value (mean) of each parameter and the values for external inputs (Matrix C). All parameters are in Hz.

Finally, we investigate how the bilinear and W-C models inform us about the microcircuitry of the MNS. For this, we perform a BMA analysis for both Bilinear and W-C single-state models over all model space, 540 models. Inspection of Figure 7 and Tables 2, 3 show the parameter estimations for the two models are very different from each other. In Figure 7, only the parameters with a posterior probability of greater than 0.95 (*P* > 0.95) are shown. For both models, the self-inhibitory connections and the forward connections from STS to BA44 and IPL are in common; however, the W-C model connections appear stronger. Regarding the model differences, for the W-C model, there are reciprocal connections between BA44 and IPL with modulatory inputs on all significant intrinsic connections except for IPL → BA44 (however, its probability is very close to 0.95). In addition, the bilinear model predicts the BA44 → IPL as weak inhibitory connection and the W-C model as an excitatory and robust connection.

**TABLE 2:** BMA results (matrix B).

Bilinear from to	STS	IPL	BA44
STS	−0.4772 (1.00)	−0.0671 (0.67)	−0.1750 (0.88)
IPL	0.3841 (0.99)	−0.4731 (1.00)	−0.0113 (0.53)
BA44	0.3953 (0.99)	0.0452 (0.62)	−0.4776 (1.00)

Wilson-Cowan			

STS	−0.4919 (1.00)	−0.1748 (0.82)	−0.1431 (0.77)
IPL	0.9006 (1.00)	−0.4795 (1.00)	0.6591 (0.99)
BA44	1.1071 (1.00)	0.5788 (0.99)	−0.4798 (1.00)

**The expected values (mean) for endogenous connectivity (Matrix A) (in Hz). In parentheses, the posterior probability is shown for each parameter to be different from zero.**

**TABLE 3:** BMA results (matrix B).

Bilinear from to	STS	IPL	BA44
STS	–	−0.0005 (0.50)	−0.0144 (0.59)
IPL	0.0853 (0.85)	–	0.0386 (0.70)
BA44	0.0989 (0.87)	0.0433 (0.73)	–

Wilson-Cowan			

STS	–	0.0094 (0.54)	0.0095 (0.54)
IPL	0.1711 (0.96)	–	0.1609 (0.96)
BA44	0.2059 (0.97)	0.1515 (0.947)	–

**The expected values (mean) for modulatory connectivity (Matrix B). In parentheses, the posterior probability is shown for each parameter to be different from zero.**

### Validation of Synthetic Data

**FIGURE 8:**
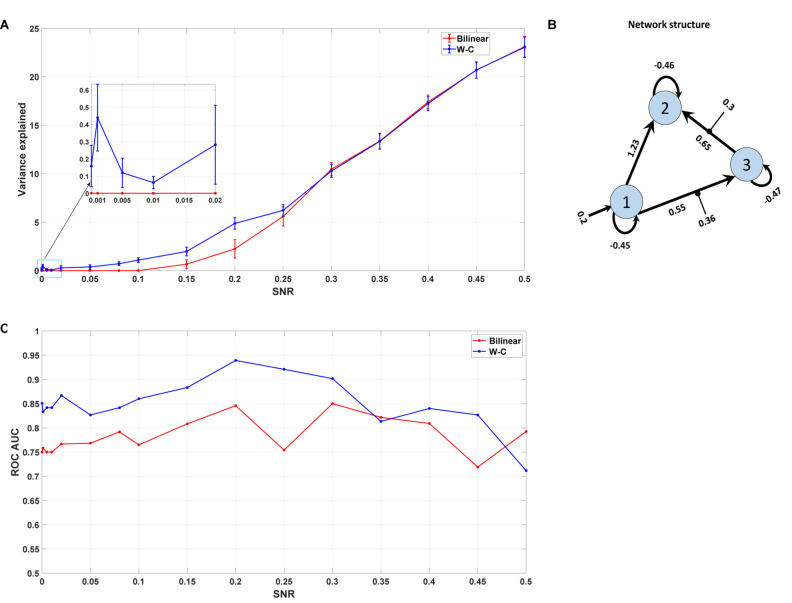
Synthetic data result. **(A)** The mean value of the variance explained by the model with different Signal to Noise Ratios for both W-C and bilinear models (10 realizations of noise for each SNR). Error bars are standard errors. **(B)** The underlying model’s network structure, which is used to generate synthetic data with the estimated parameters (the mean of expected values) from the novel empirical data. **(C)** Area under the curve (AUC) of the receiver operator characteristic (ROC) curves of detecting the existence of a connection between two areas as a function of the SNR value.

Here, we are interested in investigating the robustness of the modified single-state DCM with a synthetic data set for which the properties are known. We generated the synthetic fMRI data using the standard bilinear equation for a three-area model achieved by analyzing real data and adding random noise with different signal-to-noise ratios (see section “Materials and Methods”). In this way, we could check how well the modified DCM could identify the underlying “ground truth” based on synthetic data with different noise levels. In this regard, we compared the Wilson-Cowan model with the bilinear model by checking the percentage of variance explained by the model for different SNRs. As can be seen in Figure 8A, even for very small values of SNR (zooming portion), the W-C model fits the data better than the bilinear model in a significant way (*P* = 0.013). With increasing the SNR values, W-C models still fit the data better (but not significantly, *P* = 0.42) until explained variance values merge again for larger SNR. This shows how the W-C model enables an inversion scheme to escape the local minima in a low to a higher SNR range. In Figure 8A, we also add error bars to show that the explained variance is actually (significantly) different for intermediate SNRs. We roughly estimate the SNR for the novel empirical data used in the previous section to be 0.02 (Hadi, 2014), being at the lower end of the spectrum.

As any sensitive measure can be prone to produce false positive results, we assess the probabilities for each connection in the reconstructed model and compare the results with the “ground truth” network structure that was used to generate the synthetic data (Figure 8B). In particular, we vary the threshold p the probability for a connection needs to exceed to predict that particular connection to exist. If such a predicted connection does not exist in the generating model, it is counted as a false positive. Conversely, any missing connection in the reconstructed model that is present in the generating model is considered a false negative. Plotting the percentage of true positives (1 minus false negatives) against the percentage of false positives yields a receiver operator characteristic (ROC) curve, and the area under this curve (AUC) is a measure of the diagnostic ability of the model independent of the choice of the threshold. Figure 8C shows the AUC of the ROC curves for different signal-to-noise ratios. It is apparent that the W-C model is more efficient in correctly detecting connections between the areas than the bilinear model for small SNR values (below 0.3), while the detection performance of the two models converges for larger SNR values. In particular, false positive rates are comparable for both models at all SNR values, while false negative rates are lower for the W-C model at low SNR values.

## Discussion

In this paper, we present a new DCM variant for fMRI on the level of neuronal states in which the equations have a sigmoidal form for the latent variables. Using our measurements as well as synthetic and established real data, we show by Bayesian model comparison that the modified model explains better with the observed data than the standard bilinear equation and allows us to detect smaller effects. Furthermore, our results support current theories on information flow in the MNS.

In particular, Bayesian Model Selection showed that the Wilson-Cowan-based models with the sigmoidal form have more significant expected and exceedance probabilities than the standard bilinear equation models for single state DCM. Moreover, at the microcircuitry level, it informs us more about the network connections responsible for imitation in the MNS. Thus, we have shown that DCM with a slightly more complex neuronal model outperforms the simple linear model, and additionally allows us to make predictions about local neuronal circuits, namely on the slope of the input-output relation. Furthermore, 18 participants out of all 42 subjects gave a flatline in the predicted time series with the bilinear models. With the Wilson-Cowan models, 17 of these participants could be rescued for analyses. Our significance threshold (*p* < 0.5) for time series extraction was untypically lenient for DCM analyses. The Wilson-Cowan modification might be a way to fix the limitation of DCM to results with large effect sizes. While the possibility of false-positive results due to the more lenient threshold should be considered, results from synthetic data suggest that false positive rates are similar for Wilson-Cowan and bilinear DCM at all levels of noise.

We also implemented the modified model in the two-state DCM, where each region consists of excitatory (glutamatergic) and inhibitory (GABAergic) subpopulations (Marreiros et al., 2008a), just like the model proposed by Wilson and Cowan (1972). By performing the Bayesian model selection for the empirical data on imitation and comparing it with a single-state model, we found that the single-state model fits better to data, in opposition to the report in the original paper (Marreiros et al., 2008a). However, in both cases (single- and two-state), the Wilson-Cowan model reached a higher probability than the standard bilinear models. One may claim that in our modification, the number of parameters has increased and so it would be trivial to have a better fit in the case of higher complexity. However, as we showed in Figure 5, we received a worse probability in the two-state analysis. Here the number of estimated parameters increases, as we also estimate all the parameters for connecting inhibitory and excitatory neurons in the two-state model. Thus, increasing the number of parameters alone does not explain the better model fit in data sets.

### Comparison to Other DCM Extensions

Since its introduction in 2003, DCM for fMRI has received several extensions and methodological refinements (Daunizeau et al., 2011; Stephan and Roebroeck, 2012; Razi and Friston, 2016). These extensions include (i) two-state DCMs (Marreiros et al., 2008a), with separate excitatory and inhibitory populations within each brain region, (ii) non-linear DCMs with a quadratic state allowing the activity of one region to modulate the connectivity between two other regions (Stephan et al., 2008), (iii) stochastic DCMs which account for random endogenous fluctuations in the neuronal states and inputs (Daunizeau et al., 2009; Li et al., 2011), and (iv) spectral DCMs for modeling resting-state fMRI data, which estimate the covariance of the hidden states instead of the states itself (Friston et al., 2014; Razi et al., 2015). Furthermore, for the large scale brain regions, a linear DCM in the frequency domain using regression DCM (Frässle et al., 2017) has been developed for the task-related fMRI, and in this way, compute for hundreds of regions is now possible (Frässle et al., 2018).

There have been some advances in hemodynamic transfer function (HRF) of the DCM for fMRI that resolves the limitation for the decoupling of BOLD signal and Cerebral blood volume (CBV) (Havlicek et al., 2015), which cannot be achieved with the standard hemodynamic model in DCM (Stephan et al., 2007b)- and also DCMs with laminar resolution measured with high-resolution fMRI (Heinzle et al., 2016). However, in this study, we use the original hemodynamic model (Stephan et al., 2007b) for consistency with the framework mostly used in DCM studies.

Common to all these DCM variants for fMRI is the relative simplicity of the neuronal state equations that utilize parameters that are far from the underlying biology and difficult to match with parameters used in other fields of computational modeling of the brain. On the other end of the spectrum, DCM variants employed for EEG/MEG (David et al., 2006; Kiebel et al., 2008; Moran et al., 2013) and very recently also for fMRI (Friston et al., 2019; Wei et al., 2020) include complex neuronal state equations consisting of currents and membrane potentials instead of firing rates (Marreiros et al., 2008b; Friston et al., 2019; Wei et al., 2020) with up to four interacting subpopulations in each region representing different cell type (Bastos et al., 2015; Friston et al., 2019). The purpose of this kind of DCM is to make use of both EEG and fMRI data by feeding the fMRI inversion with posteriors estimated from EEG data (Jafarian et al., 2020; Wei et al., 2020). However, considering these complex models only for fMRI data may come at the cost that the numerous parameters of these models are much harder to fit the limited amount of data. While it has been argued that the limited temporal resolution of fMRI data can be compensated by spectral information (Friston et al., 2019), such estimations may also be limited by the strong and diverse fluctuations in the data that cannot easily be attributed to neuronal and hemodynamics sources. The present DCM variant with the Wilson-Cowan equation can compromise between very simple and very complex neuronal state equations. It is easy to implement and opens the possibility of inferring the global input-output properties of the neurons within each region. These properties can be compared to the f-I curves that are measured in electrophysiological recordings of single neurons via data-driven network simulations of a single region (Hass et al., 2016). Moreover, the Wilson-Cowan form of DCM has increased the interpretability of the connection matrices (A, B, and C), and now they can be directly interpreted. Below, we elaborate on how the DCM results can be used to constrain local networks, and thus allow inferring about the properties of the involved neurons.

### The Role of Non-linearity

The currently established version of the non-linear DCM (Stephan et al., 2008) was achieved with the further Taylor extension of Eq. 1 to the quadradic term and defining an additional matrix D for the modulatory inputs from regions. However, the version presented in this study is a formally motivated approach to non-linearity. Rather than approximating the non-linear activation function by a Taylor expansion, the Wilson-Cowan equation assumes a particular form for this function, namely a sigmoid function shown in Figure 1. This function is chosen to mimic the input-output relation in real neurons and cannot be emulated by a quadratic term. Additionally, the Wilson-Cowan equation adds a dynamic component in the form of relaxation to a steady-state given by the output of the sigmoid with a specific time constant. However, we found that including this time constant did not contribute much to the model evidence, probably due to the limited time resolution, so we removed this parameter and concentrated on the non-linearity.

If the input to a given region is close to zero, the sigmoid function is almost linear (Figure 1). In this regime, the extended DCM does not react qualitatively different from the bilinear model. One could argue that the slope parameter is redundant in this regime, as any change in excitability could be compensated by the inverse change in the connection matrix A. However, as the (absolute) input increases (i.e., during the experimental conditions), the non-linearity of the sigmoid function extends its influence, ultimately leveling off the impact of the input and saturating the output into a limited maximum. This kind of non-linearity mimics the limited dynamic range of neurons, physiologically incorporated in the form of a depolarization block (Dayan and Abbott, 2001). In the model, the dynamic range of a region is governed by the sigmoid slope: A large slope implies a narrow range, a smaller slope widens this range (Figure 1). Introducing a limited dynamic range adds an important degree of freedom to the DCM: In a linear neuronal state equation, a large input can lead to a destabilization of the entire system; thus, the strength of the connections is strongly constrained. For this reason, the behavior of the system must be constrained, e.g., by using shrinkage priors or limitations on the sign of the connectivity matrix. Furthermore, the limited input strength can potentially prevent sufficient differentiation between resting and active states. On the other hand, in a Wilson-Cowan-type model, the effect of the input is intrinsically limited by the non-linearity, so even strong inputs will not destabilize the system, allowing for much more flexibility e.g., choosing priors that reflect knowledge about the connectivity structure. In summary, the linear model approximates more complex dynamics close to a fixed point (given by the resting state) and can thus be destabilized if it is driven away from this fixed point. In contrast, in the Wilson-Cowan model, external input creates a new stable fixed point at the higher activity. In the two-state DCM, the Wilson-Cowan model exhibits an even richer dynamic repertoire, including the possibility of persistent activity and oscillations at various frequencies (Wallace et al., 2011). Thus, the present extension of DCM considerably extends the range of dynamic behaviors with only a small increase in model complexity.

### Implications for the Mirror Neuron System

Mirror neurons have first been found in the monkey brain and have been repeatedly shown to respond to both executed and observed actions (di Pellegrino et al., 1992; Rizzolatti and Fogassi, 2014). The problem that we encounter is that while mirror neurons are a highly promising candidate to allow interpersonal understanding, we can hardly measure them in the human brain. Thus, studies in humans mainly rely on fMRI or other indirect techniques. For observation and execution of actions, including imitation of facial expressions (Iacoboni et al., 2001, 2005; Carr et al., 2003; Mier et al., 2010a), studies show activity in regions that are homologs of the primate brain areas linked to mirror neurons, namely in inferior prefrontal gyrus (IFG), inferior parietal cortex (IPL), as well as in superior temporal sulcus (STS), a region of highest importance for action perception, but without own motor neurons. There is only one study directly measuring neurons with such a mirror property (Mukamel et al., 2010), and the patients examined had surgeries mainly outside those central regions of the MNS. While the authors showed the activity of neurons in the temporal lobes and further brain regions that are active during observation and execution of actions, more studies are needed to get a deeper understanding of the physiology and functioning of mirror neurons in the human brain. To date, these properties of the mirror neurons are missing even in the monkey literature. Using a two-stage modeling approach (see below) is a way to get closer to the neuronal activity underlying the BOLD signal linked to such a mirroring process in humans.

In a first step, we showed through the empirical fMRI data that the modified model has a higher probability and validate this with synthetic data based on the real data for different values of the signal to noise ratio. The empirical fMRI data on the imitation of emotional facial expressions shows connectivity between STS, IPL, and BA 44. Based on prior knowledge, the driving input was fixed to the STS (Iacoboni et al., 2001; Kilner et al., 2007). The model presented in Figure 7, from the BMA result on W-C DCM, showed a connection from STS to BA 44 and IPL with mutual coupling between BA 44 and IPL. It can be assumed that STS provides visual input, the IPL codes the exact motor action while BA44 codes the motor goal. In the context of predictive coding, this feedback from BA 44 to IPL is used for updating the motor action with the motor goals (Kilner et al., 2007). Thus, our results are in good agreement with the assumed function of STS, BA 44, and IPL for motor imitation and add empirical evidence for effective connectivity between these regions for imitating facial expressions.

### Constraints of Local Neural Networks

While the focus of this paper is the introduction of a non-linear DCM variant, the ultimate goal of the underlying project is a two-stage modeling approach which uses the results from DCM to constrain an existing, completely data-driven spiking network model (Hass et al., 2016) to construct a computational model of the human MNS. The spiking network model has previously been shown to be a statistically accurate description of rodent neural activity *in vivo*. However, the parameters of the model were adjusted exclusively by *in vitro* anatomical and electrophysiological data (Hass et al., 2016). In this way, the non-linear extension of the DCM technique presented here allows combining local modeling with constraints from animal experiments and global modeling with constraints from fMRI data. To account for the potential differences in species and brain regions, we introduced a number of global scaling parameters for the neural and synaptic properties, which are being adjusted to the DCM results (Sadeghi et al., 2017). These adjustments will lead to a model with unprecedented predictive power about the physiological properties and the temporal dynamics of the human MNS, as it accounts both for the global dynamics in humans obtained from DCM and for the detailed neuronal machinery on the level of local circuits, which are likely to be conserved across species. In contrast to existing, more abstract models of the MNS (Oztop et al., 2006; Triesch et al., 2007; Caligiore et al., 2010; Sasaki et al., 2012; Thill et al., 2013), this model holds the promise to capture detailed neuronal phenomena such as the suppression of the mu rhythm (Moore et al., 2012; Hobson and Bishop, 2016) or the modulation of the MNS by neurotransmitters (Durstewitz et al., 2000; Hass and Durstewitz, 2011; Meyer-Lindenberg et al., 2011).

## Data Availability Statement

The datasets for this article are not publicly available because of the data protection reasons for the fMRI-data of the imitation task. Requests to access the datasets should be directed to SNS, stephanie.3.schmidt@uni-konstanz.de.

## Ethics Statement

The studies involving human participants were reviewed and approved by local ethics board at the Medical Faculty Mannheim, University of Heidelberg (2015-501N-MA). The patients/participants provided their written informed consent to participate in this study.

## Author Contributions

JH designed and directed the study. DM and SNS designed and performed the experiments and analyzed the fMRI data. SS performed the DCM analysis, drafted the manuscript, and designed the figures in consultation with JH and DM. SS and JH interpreted the results. MG aided in DCM analysis and interpreting the results. All authors contributed to the manuscript revision, read, and approved the submitted version.

## Conflict of Interest

The authors declare that the research was conducted in the absence of any commercial or financial relationships that could be construed as a potential conflict of interest.

## Footnotes

1 http://www.fil.ion.ucl.ac.uk/spm/software/spm8/
